# Expression, Immobilization and Enzymatic Properties of Glutamate Decarboxylase Fused to a Cellulose-Binding Domain

**DOI:** 10.3390/ijms13010358

**Published:** 2011-12-28

**Authors:** Hyemin Park, Jungoh Ahn, Juwhan Lee, Hyeokwon Lee, Chunsuk Kim, Joon-Ki Jung, Hongweon Lee, Eun Gyo Lee

**Affiliations:** Biotechnology Process Engineering Center, KRIBB, Daejeon 305-600, Korea; E-Mails: gohworld@kribb.re.kr (H.P.); ahnjo@kribb.re.kr (J.A.); jhlee@kribb.re.kr (J.L.); tntn7616@kribb.re.kr (H.L.); chskim@kribb.re.kr (C.K.); jkjung@kribb.re.kr (J.-K.J.); hwlee@kribb.re.kr (H.L.)

**Keywords:** GAD, cellulose-binding domain, fusion protein, immobilization

## Abstract

*Escherichia coli*-derived glutamate decarboxylase (GAD), an enzyme that catalyzes the conversion of glutamic acid to gamma-aminobutyric acid (GABA), was fused to the cellulose-binding domain (CBD) and a linker of *Trichoderma harzianum* endoglucanase II. To prevent proteolysis of the fusion protein, the native linker was replaced with a S_3_N_10_ peptide known to be completely resistant to *E. coli* endopeptidase. The CBD-GAD expressed in *E. coli* was successfully immobilized on Avicel, a crystalline cellulose, with binding capacity of 33 ± 2 nmol_CBD-GAD_/g_Avicel_ and the immobilized enzymes retained 60% of their initial activities after 10 uses. The results of this report provide a feasible alternative to produce GABA using immobilized GAD through fusion to CBD.

## 1. Introduction

Glutamate decarboxylase (GAD, EC 4.1.1.15) catalyzes the conversion of glutamic acid to gamma-aminobutyric acid (GABA) by consuming one intracellular proton and pyridoxal 5'-phosphate (PLP) as a cofactor and producing CO_2_. GADs are ubiquitously found throughout the biological world from microorganisms to mammals [1]. GABA plays important physiological roles such as an inhibitory neurotransmitter with hypotensive, diuretic, and analgesic effects in the mammalian central nervous system [2–4], and is also involved in an acid-tolerance mechanism in microorganisms [5].This molecule can also be easily lactamized into 2-pyrrolidone, the precursor of nylon 4 [6,7], which has brought attention to GAD as a key component of GABA production through an enzymatic process.

Among microbial GADs, *E. coli*-derived GAD (EcGAD) is a well-characterized enzyme [8–11]. EcGAD possesses some superior enzymatic characteristics including a high affinity for substrate and high reaction rate compared to other GADs [1,4]. Some studies have focused on potential for industrial applications of EcGAD by improving its enzymatic properties and reaction conditions [6,9,12]. However, few studies have been performed on the immobilization of EcGAD for reuse [7]; in particular, there is no report of experiments using an affinity tag.

Most cellulose-degrading enzymes contain three domains: a cellulose-binding domain (CBD), a flexible linker region, and a catalytic domain. [13–15]. The catalytic and CBDs, connected by a flexible polypeptide linker, participate in hydrolyzing and binding to cellulose, respectively. Some CBDs have been developed as a versatile tag for affinity procedures due to their specific and irreversible cellulose-binding capabilities. Cellulose is relatively cheap, chemically inert, commercially available in many different forms and has a low non-specific affinity for most proteins [14,16–18].

In this study, EcGAD was fused to the CBD from *Trichoderma harzianum* endoglucanse II (THEG). The GAD activity and cellulose-binding property of the fusion protein were investigated. Moreover, immobilization of this construct to a cellulose matrix was evaluated.

## 2. Results and Discussion

### 2.1. Expression of GAD Fused to CBD in *E. coli*

**Figure 1:**
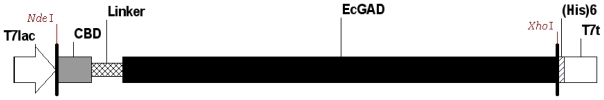
A schematic representation of the expression vector for CBD-GAD-H6 fusion protein. *E. coli*-derived glutamate decarboxylase (EcGAD), glutamate decarboxylase from *E. coli*; CBD and linker, the cellulose-binding and linker domains of *T. harzianum* endoglucanase II; (His)_6_, 6× histidine tag sequence; T7*lac*, T7 promoter sequence; T7t, T7 terminator sequence.

**Figure 2:**
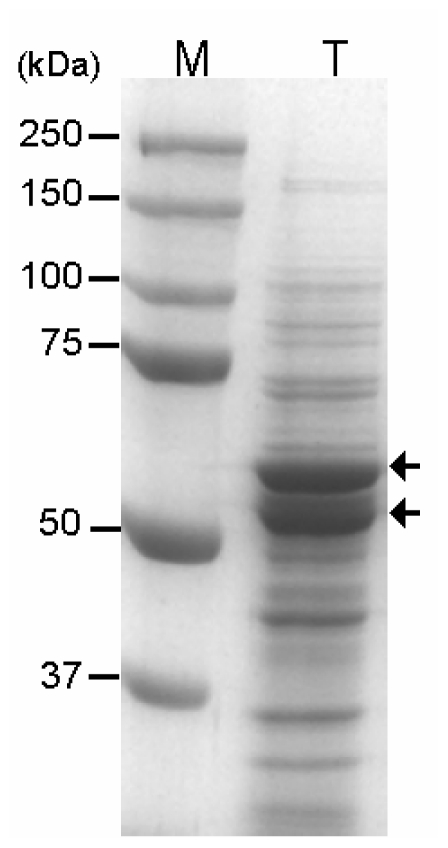
Expression of GAD fused to CBD in *E. coli*. Lane M, molecular weight (Mw) marker; lane T, total protein from the induced cells. The arrows indicate the position of the expressed GAD proteins.

For the fusion of EcGAD with CBD, the pEKPM-CBD-Lk-EcGAD-H6 expression vector was constructed (Figure 1). In this vector, EcGAD was fused to CBD and native linker of *T. harziaum* endoglucanase II [19] at its *N*-terminus and six histidine at its *C*-terminus, and the fused gene was placed under the control of a T7 promoter. GAD was expressed in *E. coli* BL21(*DE3*) harboring pEKPM-CBD-Lk-EcGAD-H6 through IPTG induction. As shown in Figure 2, two distinct bands were observed by SDS-PAGE, indicating that the fusion protein was partially degraded. *N*-terminal amino acid sequence analysis confirmed that proteolytic cleavage occurred in linker region (-ATTM↓STTT-, ↓ indicates cleavage site). This phenomenon might be related to the fact that inter-domain linkers are particularly susceptible to proteolysis [12]. The reason is that flexible regions can more easily adopt conformations compatible with the active site of endopeptidase [12].

### 2.2. Modification of the Linker Peptide Connecting CBD with GAD

**Figure 3:**
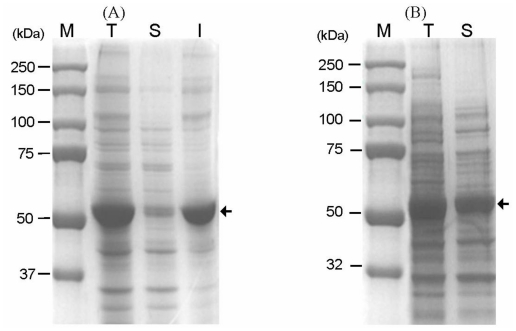
Expression of GAD fused to CBD with a modified linker in *E. coli* at 37 °C (**A**) and 18 °C (**B**). Lane M, Mw marker; lane T, total protein from the induced cells; lane S, soluble fraction from the induced cells; lane I, insoluble fraction from the induced cells. The arrows indicate the position of the CBD-GAD-H6 protein expressed in *E. coli*.

In order to prevent proteolytic cleavage of the linker peptide, a native linker peptide was replaced with a S_3_N_10_ peptide known to completely resist *E. coli* endopeptidase [12]. As shown in Figure 3A, the fusion protein with a S_3_N_10_ linker was successfully expressed without being degraded by proteolysis, which was confirmed by *N*-terminal amino acid sequencing. However, analysis of the cellular fraction from induced cells showed that most of the fusion proteins were expressed as an insoluble form. To increase the solubility, the transformants were cultured at a low temperature (at 18 °C) and gene expression was induced by lactose (Figure 3B).

### 2.3. Immobilization of CBD-GAD to the Cellulose Matrix

**Figure 4:**
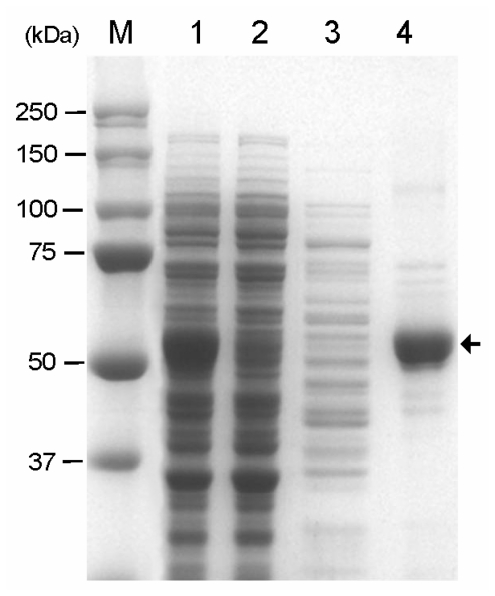
Purification of CBD-GAD using His-tag affinity chromatography. Lane M, Mw marker; lane 1, soluble fraction from the induced cells; lane 2, flow-through fraction; lane 3, washed fraction; lane 4, purified CBD-GAD. The arrow indicates the position of the purified CBD-GAD-H6 protein.

**Figure 5:**
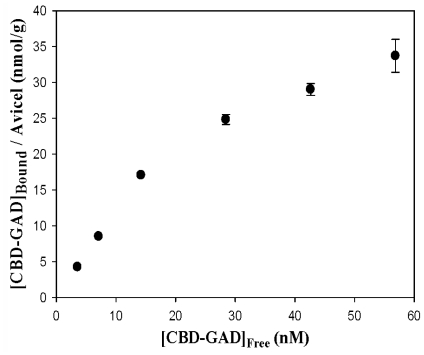
Adsorption isotherm for binding of CBD-GAD to Avicel. Adsorptions were evaluated in 20 mM sodium acetate buffer at pH 6.0 and room temperature. [CBD-GAD]_Bound_ and [CBD-GAD]_Free_ are the concentration of bound enzyme (nmol_CBD-GAD_/g_Avicel_) and free enzyme (nM), respectively.

**Figure 6:**
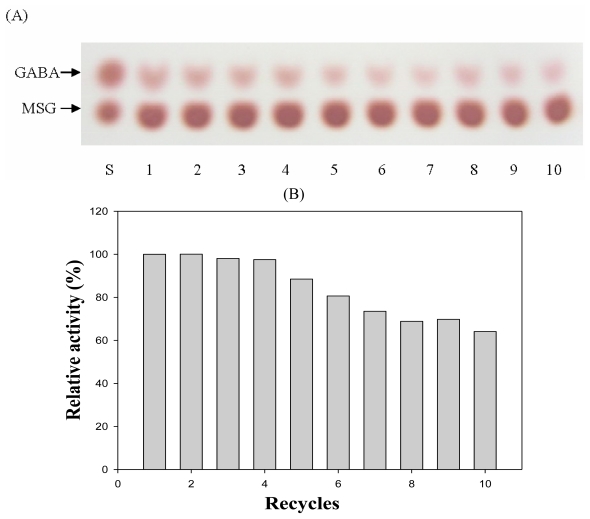
Thin layer chromatography (**A**) and relative activity (**B**) of GAD immobilized on Avicel after multiple uses. After the enzyme reaction was preceded at 37 °C for 30 min at pH 4.0, the immobilized enzyme was recovered by centrifugation and repeatedly re-used. The Avicel recovery was estimated to be about 99.7% at each step. S, standard for GABA and monosodium glutamate (MSG); number, recycled times.

The soluble CBD-GAD fusion protein was purified using a Ni-NTA column and eluted by a stepwise gradient of imidazole. The imidazole fraction (250 mM) contained a 58 kDa protein corresponding to CBD-GAD with a purity of 84% (Figure 4). The specific activity of the purified CBD-GAD was 24.44 ± 0.77 U/mg, which was lower than the GAD fused to six histidine (35.01 ± 1.03 U/mg) expressed in *E. coli* in this study. The purified enzyme was used to evaluate the adsorption isotherm of CBD-GAD on Avicel (Figure 5). The cellulose-binding capacity was calculated to be 33 ± 2 nmol_CBD-GAD_/g_Avicel_; this was relatively low compared to other CBD-fusion proteins (0.082–1.10 μmol/g) reported in previous studies [20–22]. This low binding capacity might have been attributed to be due to steric hindrance by the use of modified linker or the complex nature of CBD-GAD. The reusability of the GAD immobilized on Avicel was determined by comparing its relative activity after several uses. As shown in Figure 6, the immobilized GAD retained about 60% of its initial activity after ten consecutive uses.

## 3. Experimental Section

### 3.1. Bacterial Srains

*E. coli* DH5α [F^−^, φ80d*lac*ZΔM15, *end*A1 *hsd*R17(rK^−^, mK^+^), *sup*E44, *thi-*1, λ^−^, *rec*A1, *gyr*A96] was used as the host strain for cloning and plasmid maintenance. *E. coli* BL21 [F^−^, *hsd*S, *gal*, *omp*T, rB^−^, mB^−^] (Novagen, USA) harboring a lambda derivative, *DE3*, was used for the expression of GAD.

### 3.2. Plasmid Construction

For the efficient cloning work and use of kanamycin as a selective marker, DNA region between *Bgl*II and *Nde*I in pET-28b(+) was replaced by the region between same enzyme sites in pET-22b(+), which created a modified pET-28b(+). For the CBD-GAD fusion, the gene encoding CBD and linker of THEG were amplified from pGAL-CBD-Lipase [19] using a forward primer (5′-GAAGGAGATATACATATGCAGCAAACTGTTTGGGGGCAA-3′) and a reverse primer (5′-ACGGAGCTCGAATTCGGATCCAGAGCTAGTTGGCGGAGTAGC-3′) in which the underlined characters were introduced for in-fusion ligation. The polymerase chain reaction (PCR) product was ligated into the modified pET-28b(+) plasmid (Novagen, USA) digested with *Nde*I and *BamH*I using the In-fusion™ Advantage PCR cloning kit (Clontech, USA). The resulting plasmid was named pEKPM-CBD. The GAD gene was amplified from *E. coli* genomic DNA by PCR using a forward primer (5′-ACTCCGCCAACTAGCTCTGGATCCGATAAGAAGCAAGTAACG-3′) and a reverse primer (5′-GTGGTGGTGGTGGTGGTGCTCGAGGGTATGTTTAAAGCT-3′) in which the underlined base pairs were introduced for in-fusion ligation. The PCR product was ligated into the pEKPM-CBD plasmid previously digested with *Bam*HI and *Xho*I using the In-fusion™ Advantage PCR cloning kit, which created pEKPM-CBD-Lk-EcGAD-H6. To replace the native linker peptide with S_3_N_10_ linker sequence as suggested by Kavoosi *et al.* [12], CBD with the S_3_N_10_ linker are amplified from pEKPM-CBD-Lk-EcGAD-H6 using the specific primers (forward: 5′-GAAGGAGATATACATATGCAGCAAACTGTTTGGGGG-3′, reverse: 5′-CTTCTTATCGGATCC GTTGTTGTTGTTGTTGTTGT TGTTGTTGTTGCTGCTGCTAATGCATTGAGCGTAGTA-3′ in which the underlined characters were introduced for in-fusion ligation). The PCR products containing CBD and the S_3_N_10_ linker were ligated into pEKPM-CBD-Lk-EcGAD-H6 previously digested with *Nde*I and *Bam*HI, which created pEKPM-CBD-mLk-EcGAD-H6.

For GAD protein without CBD, the GAD gene was amplified from *E. coli* genomic DNA by PCR using a forward primer (5′-GAAGGAGATATACATATGGATAAGAAGCAAGTAACG-3′) and a reverse primer (5′-GTGGTGGTGGTGGTGGTGCTCGAGGGTATGTTTAAAGCT-3′) in which the underlined base pairs were introduced for in-fusion ligation. The PCR product was ligated into the modified pET-28b(+) plasmid previously digested with *Bam*HI and *Xho*I using the In-fusion™ Advantage PCR cloning kit, which created pEKPM-GAD-H6.

### 3.3. Expression of CBD-GAD

Transformants were cultured overnight in a test tube containing 2 mL of Luria-Bertani (LB) media with kanamycin (50 μg/mL) at 37 °C. The culture was then transferred to 1 L baffled flasks containing 200 mL of LB media supplemented with kanamycin (50 μg/mL). When cell density reached an optical density (OD) of 0.5 at 600 nm as measured by a spectrophotometer (UVICON, Switzerland), induction was initiated with 0.4 mM IPTG for 3 h. After IPTG induction, the cells were harvested and lysed by sonication for further analysis. Proteins in the lysates were separated by SDS-PAGE according to the method by Laemmli [23] on 10% polyacrylamide gels using a mini-gel apparatus (Hoefer, USA). The separated proteins were stained with Coomassie blue. For culturing at low temperature, the transformants were cultured using the protocol described above at 37 °C to an OD of 0.5 and induced overnight with 0.1% (w/v) lactose.

### 3.4. *N*-Terminal Amino Acid Sequencing

CBD-GAD-H6 was isolated using SDS-PAGE, and the corresponding bands were transferred to a PVDF (polyvinylidene fluoride) membrane using a semi-dry electroblotter (TE 77 PWR, Amersham Bioscience, UK). The membrane was stained with Ponceau S (AMRESCO, USA) to visualize the GAD bands and *N*-terminal amino acid sequencing was performed by the Korea Basic Science Institute (Republic of Korea).

### 3.5. Purification of CBD-GAD and GAD Protein

The transformants were harvested by centrifugation. The cells were then resuspended in B-PER^®^II reagent (bacterial protein extraction reagent, Pierce, USA) to extract soluble proteins. The supernatant was collected for purification; nickel affinity chromatography using Ni-NTA resin (HisTrap, GE Healthcare, UK) was performed to purify CBD-GAD and GAD proteins. The bound proteins were eluted with a three-step gradient of imidazole (12.5, 25, and 250 mM) in 20 mM sodium phosphate buffer with 0.5 M NaCl, pH 7.4. Fractions collected at each step were analyzed by 10% SDS-PAGE. Purified proteins were dialyzed overnight against 20 mM sodium phosphate buffer (pH 7.4) at 4 °C.

### 3.6. Assay of GAD Activity

Enzymatic activities were measured by a 718 Stat Titrino (Metrohm, Switzerland) with an aqueous solution of 0.1 M HCl. The solution containing MSG of 0.1M and PLP of 0.02 mM was adjusted to final pH of 4.0 and an enzyme was added. The enzyme reaction was performed at 37 °C while recording the titration. Activities were calculated by the slope of the titration curve over 5 min. One unit was defined as the amount of enzyme required for being added 1 μmol H^+^ per minute under assay conditions.

### 3.7. Adsorption Isotherm Measurements

To estimate the binding capacity of CBD-GAD attached to the Avicel, adsorption isotherm measurements were taken at room temperature in 1.5 mL Eppendorf tubes. Samples containing 0–60 nM of CBD-GAD and 0.2 mg of Avicel were mixed with 20 mM sodium acetate buffer (pH 6.0) in a final aqueous volume of 1.2 mL. Each solution was incubated for 1 h to allow the adsorption to saturate. The supernatants were collected by centrifugation and the protein concentration was measured using a BCA protein assay kit (Pierce, USA). This value was then subtracted from the initial amount of protein added to the reaction tube and the difference was considered to be amount of proteins adsorbed onto Avicel. All values were measured in triplicate.

### 3.8. Reusability Assay of Immobilized GAD

Relative quantification of retained activities after use of immobilized GAD was performed by TLC combined with image analyzer. Samples were prepared as follows; Enzyme was added into the 100 mM MSG and 0.02 mM PLP in 20 mM sodium acetate buffer (pH 4.0). After enzyme reaction at 37 °C for 30 min, immobilized enzyme was recovered by centrifugation and re-used after washing with sodium acetate buffer (pH 4.0). The loss of enzyme molecules due to the recovery process was calculated as 0.3% at each step. The supernatants after enzyme reaction and GABA standard solution were spotted on 10 cm × 10 cm glass TLC plates precoated with 60F254 silica gel (Merck, Germany). The mobile phase was prepared by mixing *n*-butanol/acetic acid/water (5/3/2, v/v/v). The plates were developed by applying ninhydrin reagent (Fluka, Switzerland) then dried at 80 °C for 5 min. The image analysis of the spot on the plate was processed by TotalLap TL100 Image Analysis Software (Nonlinear Dynamics, UK).

## 4. Conclusions

In conclusion, we succeeded in producing recombinant *E. coli* which can express relatively high-level of GAD fused to CBD with a histag to facilitate purification as soluble forms through the modification of linker peptide and low temperature culture condition. This bifunctional chimeric protein simultaneously showed both GAD activity and cellulose-binding ability, which allowed it to be immobilized on a cellulose matrix. To our knowledge, this is the first report on the immobilization of GAD using affinity-tag to obtain GABA by bio-conversion. Applying of low-priced cellulose as matrix and reutilizing of enzyme due to immobilization can be accompanied by decrease in cost. The presented process, therefore, can be potentially applicable to produce GABA in large-scale.
